# Physicochemical properties of dietary fiber of bergamot and its effect on diabetic mice

**DOI:** 10.3389/fnut.2022.1040825

**Published:** 2022-11-04

**Authors:** Huifan Liu, Jiaxi Liang, Churong Liang, Guiqiang Liang, Jiacong Lai, Renying Zhang, Qin Wang, Gengsheng Xiao

**Affiliations:** ^1^College of Light Industry and Food, Zhongkai University of Agriculture and Engineering, Guangzhou, China; ^2^Guangdong Provincial Key Laboratory of Lingnan Specialty Food Science and Technology, Guangzhou, China

**Keywords:** bergamot, dietary fiber, functional properties, diabetes, physicochemical effects

## Abstract

Bergamot (*Citrus medica* L. var. *sarcodactylis*) contains different bioactive compounds, and their effects remain unclear. Therefore, the structural and bio-function of bergamot dietary fiber were investigated. A sequential extraction procedure was utilized to obtain soluble dietary fiber (SDF) and insoluble dietary fiber (IDF) from bergamot. The main monosaccharide in SDF and IDF is arabinose. SDF had a porous structure, which enhanced the water and oil holding capacity, as well as the cholesterol and glucose adsorption capacity, which was superior to that of IDF. In db/db diabetic mice, SDF and IDF regulated glucose tolerance and controlled blood glucose levels. Reduction of serum total cholesterol, triglycerides, and low-density lipoprotein cholesterol in SDF and IDF could be observed. In summary, SDF and IDF from bergamot effectively promoted health in patients with diabetes.

## Introduction

Bergamot (*Citrus medica* L. var. *sarcodactylis*) belongs to the citrus family (1). It is used in China as a nutraceutical to promote health. Previous studies have revealed that functional substances found in bergamot, such as phenolic compounds, carbohydrates, and essential oils, have antioxidant (2), immunoregulatory (3), and antimicrobial effects (4).

Dietary fiber (DF), a carbohydrate-based polymer, is mostly derived from edible plants such as vegetables and fruits (5). DF is classified as soluble dietary fiber (SDF) or insoluble dietary fiber (IDF) based on its water solubility (6). Interestingly, owing to the special properties of DF, which comprises cellulose, hemicellulose, pectin, and inulin, it is indigestible and unabsorbable in the small intestine of humans (7). DF regulates the speed of chyme through the small intestines, increases stool volume, lowers total cholesterol and low density lipoprotein cholesterol levels, lowers blood glucose and insulin levels, and reduces heart disease risk (7). It was also shown that the composition and source of DF will affect its adsorption properties, such as water holding capacity and oil and cholesterol binding ability (7).

Type 2 diabetes mellitus (T2DM) is characterized by insufficient insulin production or the inability to absorb it for glycemic regulation in the human body (8). In addition, interrelated abnormalities associated with T2DM include metabolic dysregulation of proteins, lipids, and carbohydrates, hyperglycemia, insulin resistance, dyslipidemia, and persistent inflammation (9). Although various medicines such as insulin secretagogues, biguanides, and thiazolidinediones have been used to treat T2DM, these antidiabetic agents may cause side effects (10). Considering these adverse effects, studies have recently focused on plant-based polysaccharides as alternatives because of their anti-diabetic effects and low cost (10).

DF may aid in reducing appetite, dietary intake, obesity risk, blood sugar levels, and cholesterol levels (11). Gastric emptiness delay, body weight (BW) loss and postprandial glucose response control, lipid metabolism modulation, enzyme action efficacy (inhibition of -amylase and -glucosidase), and gut microbiota changes are all factors that contribute to the effects of DF on T2DM (12). The binding and excretion of bile acids by water-soluble DFs and IDFs in the small intestine is one of the primary processes behind their cholesterol-lowering properties (13). Glycemic management and insulin sensitivity in T2DM patients can be improved by consuming soluble fiber products and natural DFs, with soluble fiber products yielding better effects (14). SDF can be utilized to lower the risk of cardiovascular diseases, the glycemic response, and plasma cholesterol levels (15), protecting against diabetes. IDF has demonstrated physiological functions that include increasing feces volume, reducing intestinal transit, and suppressing pancreatic lipase activity (15).

In this study, SDF and IDF were extracted from bergamot and their physicochemical properties, water- and oil-holding capacity, cholesterol and glucose adsorption, and glycemic regulation were characterized *in vivo*. This study offers helpful information on the bioactive properties of bergamot and related DFs.

## Materials and methods

### Materials

Bergamots were obtained from Huizhou (Guangdong, China). In addition, papain, heat-stable α-amylase, cellulase, neutral protease, and saccharifying enzyme were purchased from Guangzhou Dongju Co., Ltd. (Guangzhou, China). Other chemicals and reagents used were commercially available.

### Preparation and extraction of SDF and IDF from bergamot

Bergamots were cleaned, sliced, and dried at 55°C for 24 h. Then, the dried bergamots were ground into flour in a crusher and kept at 4°C. SDF extraction was conducted as previously described (16). Briefly, acetic acid-sodium acetate buffer was stirred with the bergamot powder. The sample was heated to 100°C in a water bath for 1 h, and then it was cooled to 25°C. Cellulase with a pH of 4.9 was then added and left for 1.5 h at 50°C. The mixture was then heated for 10 min at 85°C. In a water bath, papain (0.06% *w/v*) was added to the mixture, which was then treated for 30 min at 60°C. Four liters of 95% (*v/v*) ethanol were added when the mixture was cooled to 25°C to precipitate the SDF for 12 h. The method for the extraction of IDF was also used in a previous study (16). Bergamot powder (10 g) was dissolved in deionized water (1:10 = *w:v*), centrifuged at 1,500 × g for 15 min, and the sample was precipitated. At 70°C, pH 5.5, and 0.6% enzyme dosage, α-amylase was applied to the precipitate for 80 min. To obtain the IDF, the residue was finally filtered and dried.

### Chemical composition analysis

The composition, such as ash (AOAC 920.138) and protein (AOAC 992.23), was determined as previously described (16).

### Scanning electron microscopy (SEM) analysis

The SDF and IDF were sprayed with metal, and their spatial structures were analyzed by SEM (Zeiss Supra 55, Carl Zeiss Meditec AG, Shanghai, China) at a magnification of × 200.

### Analysis of Fourier-transform infrared spectroscopy (FT-IR)

An FT-IR spectrophotometer (Bruker, Billerica, MA, USA) was used to measure the FT-IR spectra of bergamot SDF and IDF. Before being formed into pellets (5 mg DF sample/200 mg KBr) for FT-IR scanning in the 4,000–400 cm^−1^ frequency range, the bergamot SDF and IDF were ground with potassium bromide powder (KBr).

### Monosaccharide compositions

The assay for monosaccharide components was conducted as previously described by Xiong et al. with some modifications (17). Standard monosaccharides (mannose, ribose, rhamnose, glucuronic acid, galacturonic acid, arabinose, fucose, galactose, and xylose) were dissolved in distilled water to prepare reference solutions. Each sample was diluted with 2 mol/L trifluoroacetic acid (5 mL) at 120°C for 4 h. After the mixture was dried, it was dissolved in 5 mL of water to prepare the sample solutions. Then, sample solution was mixed with 0.6 mol/L NaOH and 0.4 mol/L 1-phenyl-3-methyl-5-pyrazolone (PMP) methanol solution. The mixture was incubated at 70°C for 1 h. Next, 0.3 mol/L HCl solution was used to neutralize the mixture. The mixture was extracted with chloroform (1 mL) and centrifuged three times for 10 min at 3,000 × g to obtain the supernatant. Each supernatant was then analyzed by high-performance liquid chromatography (LC-20AT, Shimadzu, Japan) with a C18 column (4.6 × 200 mm, 5 μm). The chromatographic detection conditions were 83% monopotassium phosphate (0.05 M) and 17% acetonitrile at 30°C, a flow rate of 1.0 mL/min, and a detection wavelength of 250 nm.

### Evaluation of water-holding capacity (WHC)

The WHC of the bergamot SDF and IDF was measured as described previously (18) with minor modifications. Briefly, 500 mg of sample (W1) was dissolved in 25 mL of distilled water. The mixture was then incubated for 2 h at 25°C. It was then centrifuged at 1,000 × g for 20 min to collect sediment (W2). The WHC was calculated as follows:


$$\begin{array}{l} \text{WHC}\left(\text{g}/\text{g}\right)=\left(\text{W}2-\text{W}1\right)/\text{W}1 \end{array}$$

### Oil-holding capacity (OHC)

The OHC of the bergamot SDF and IDF was determined as described previously (18) with minor modifications. A 500 mg DF sample (O1) was added to 5 mL of soybean oil with sufficient mixing, followed by 2 h of incubation at 25°C. The collected sediment (O2) was weighed after centrifugation at 1000 × g for 20 min. The OHC was determined by the following formula:


$$\begin{array}{l} \text{OHC}\left(\text{g}/\text{g}\right)=\left(\text{O}2-\text{O}1\right)/\text{O}1 \end{array}$$

### Cholesterol adsorption capacity (CAC)

The CAC of the bergamot SDF and IDF was determined using the o-phthalaldehyde method (18) with minor modifications. Egg yolk and distilled water were thoroughly mixed into a homogeneously diluted yolk solution. The 2.5 g of diluted yolk was added to the bergamot SDF (80 mg) or IDF (80 mg) sample (W). The mixture's pH was adjusted to 7.0 or 2.0, and it was then incubated for 2 h in a shaker water bath at 37°C. The blank was a diluted yolk solution without the bergamot SDF and IDF. The mixture was centrifuged at 1,000 × g for 10 min. The supernatant's absorbance was then determined at 535 nm. In the diluted yolk with bergamot SDF and IDF, the cholesterol level was measured as Cd, whereas in the diluted yolk without bergamot SDF and IDF, it was recorded as Cb. CAC was calculated as follows:


$$\begin{array}{l} \text{CAC}\left(\text{mg}/\text{g}\right)=\left(\text{Cb}-\text{Cd}\right)/\text{W} \end{array}$$

### Glucose adsorption capacity (GAC)

The glucose adsorption capacity (GAC) of the samples was evaluated as previously described (19).

### Hypoglycemic effect of DF *in vivo*

#### Mice and diet

This method was conducted as previously described (20). Briefly, 8-week-old male db/db mice and non-diabetic lean littermates (m/m) were purchased from Charles River Laboratories (Wilmington, MA, USA). After 1 week of acclimatization, the mice were randomly divided into five groups (two mice per cage, *n* = 6 in each group) for analysis: (1) non-diabetic lean littermates (m/m, NC); (2) db/db mice treated with vehicle (db group); (3) db/db mice treated with metformin 200 mg/kg BW/day (met group); (4) db/db mice treated with SDF 800 mg/kg BW/day (SDF group); and (5) db/db mice treated with IDF 800 mg/kg BW/day (IDF group). Throughout the experiments, the mice were fed a standard chow diet and tap water ad libitum. The duration of oral administration was 8 weeks. All experimental protocols were performed in accordance with the regulations of the Laboratory Animal Care and Use Committee (No. HZYX20220222641901).

Liver and pancreatic tissue samples were weighed or immediately snap-frozen in liquid nitrogen until use. The BW of each mouse was recorded every 2 weeks. The liver index was calculated according to a previous study (20). The mice were fasted overnight and anesthetized with isoflurane. Fasting blood glucose (FBG) levels were determined from the tail vein. Blood samples were collected by eyeball extirpating and centrifuged at 1,000 × g for 10 min to obtain serum. Commercial kits were used to determine the levels of glycated serum protein (GSP) and insulin according to the manufacturer's instructions (Nanjing Jiancheng Bioengineering Institute, Jiangsu, China). The homeostatic index of insulin resistance (HOMA-IR) was assessed according to the homeostasis model: HOMA-IR = FBG (mmol/L) × insulin level (mIU/L)/22.5.

#### Oral glucose tolerance test (OGTT)

Blood glucose was determined using the OGTT. Specifically, after 12 h without meals, blood samples were taken from the tail vein and the levels of glucose were evaluated. Then, glucose was given orally to the animals at a dose of 2 g/kg BW, and blood was drawn from the tail vein every 30 minutes to measure the glucose levels. The area under the curve (AUC) was calculated using the measurement results (21).

#### Analysis of lipid-lowering function

Total cholesterol (TC), triglycerides (TG), and low-density lipoprotein cholesterol (LDL-C) levels were determined using commercial reagent kits (Nanjing Jiancheng Biology Engineering Institute, Nanjing, China). All procedures were carried out by following the manufacturer's instructions.

#### Histopathological observation

Different groups of mice were used to obtain liver and pancreatic tissue samples. The tissues were dehydrated, flushed with PBS, fixed with 10% formalin, embedded in paraffin, and sectioned. Finally, the sections were stained with hematoxylin and eosin. All images were obtained using a TE 2000 fluorescence microscope (Nikon, Japan) (21).

### Statistical analysis

Data are expressed as means ± standard deviation (SD) of three replicates. All graphical representations were generated using GraphPad Prism software (version 7.0, GraphPad Software Inc., San Diego, CA, USA). The data were analyzed by Student's *t-*tests for two group, and by Tukey's multiple comparisons test with one-way ANOVA for multiple group comparisons; ^*^p < 0.05, ^**^p < 0.01, ^***^p < 0.001, ^****^p < 0.0001; NS, not significant.

## Results and discussion

### SEM analyses of SDF and IDF

**Figure 1:**
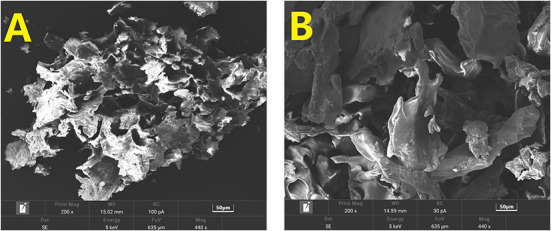
SEM analyses of SDF **(A)** and IDF **(B)** at × 200 magnification.

Images of the SDF and IDF ( × 200) were used to show the morphology and size of the SDF (Figure 1A) and IDF (Figure 1B). Unlike IDF, SDF was loosly structured with honeycomb-like holes and cracks, similar to prior work (22). The loose inner structure could be exposed by cellulase (23) and may be related to the degradation of the dense macromolecular structure due to cellulase.

In contrast, IDF was flaky and schistose, with a compact texture. However, this porous structural feature of DF is conducive to immobilizing the molecules in the network structure or holes, enhancing the binding ability of DF (19).

### FT-IR analyses

**Figure 2:**
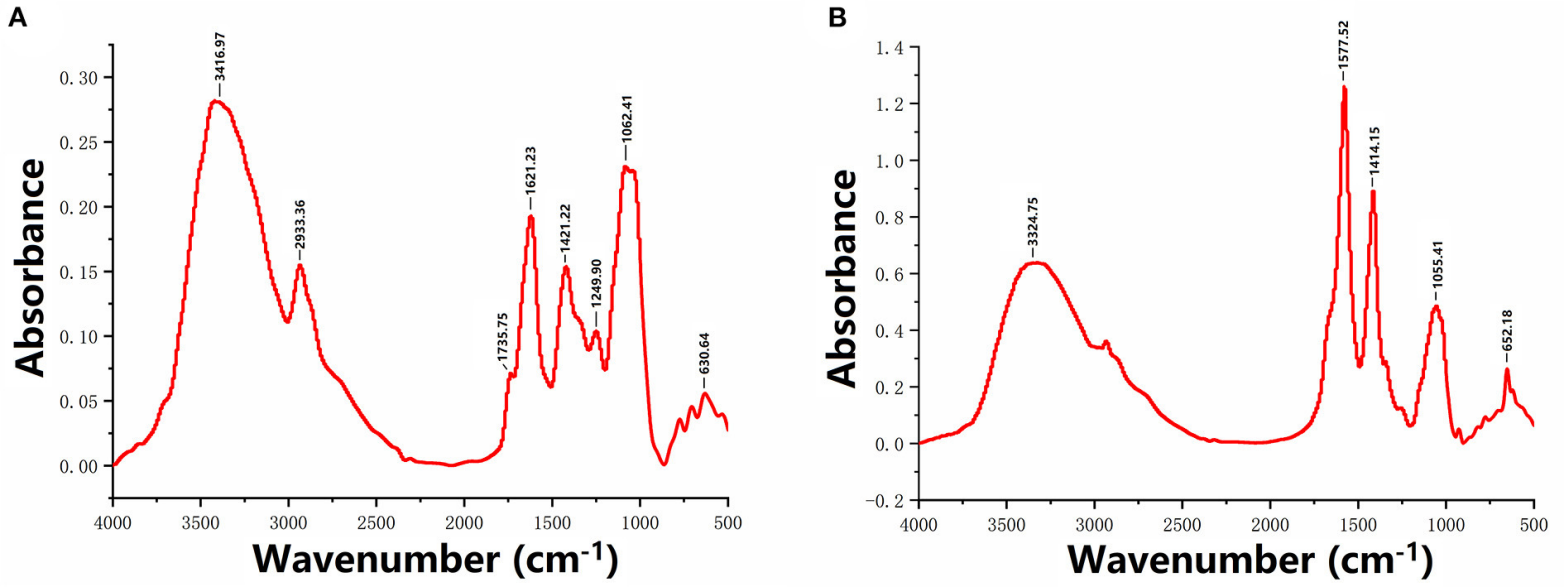
FT-IR analyses of SDF **(A)** and IDF **(B)**.

The FTIR spectra of SDF and IDF are displayed in Figure 2 to elucidate the functional groups. While several distinguishing bands in the wavenumbers of the SDF and IDF were different, their basic spectral characteristics were comparable. SDF exhibited a strong and broad absorption band at 3416.97 cm^−1^, attributed to the O-H stretching vibration of hemicellulose (24). The weak absorption bands at 2,933.36 and 1,735.75 cm^−1^ may be due to the bending and stretching vibrations of C-H (24). In addition, the peaks at 1,621.23 and 1421.22 cm^−1^ may be related to the stretching vibrations of C=O (24). This is comparable to the DF absorption bands described previously (24). Moreover, the peak at 1249.90 cm^−1^ in SDF indicates the stretching vibration of C-O (25). A signal at approximately 1062.41 cm^−1^ was observed in the spectrum of SDF, implying that the absorbance peaks were assigned to the C–O–C stretching vibration (26). Regarding IDF, most signals corresponded to those of the SDF. However, a characteristic band at 1577.52 cm^−1^ (Figure 2B) indicated C=C aromatic skeletal vibrations, arising from acetyl and ester groups. This may be attributable to lignin or hemicelluloses (27).

### Composition of SDF and IDF

The yields (g/100 g) of bergamot SDF and IDF were 38.12 ± 2.94% and 45.38 ± 3.52%, respectively. The SDF and IDF chemical compositions were analyzed (Table 1). The protein content in SDF and IDF was 7.27 ± 2.37% and 5.24 ± 0.40%, respectively. Moreover, the ash content in SDF (4.66 ± 1.43%) and IDF (3.37 ± 1.03%) was also determined. These results were consistent with previous work (18). Since bergamot had a DF content of over 30%, it might be used in food preparation as a thickening agent.

**Table 1:** Components of SDF and IDF.

	**SDF (%)**	**IDF (%)**
Yield	38.12 ± 2.94	45.38 ± 3.52
Protein	7.27 ± 2.37	5.24 ± 0.40
Ash	4.66 ± 1.43	3.37 ± 1.03

The monosaccharide compositions of SDF and IDF are shown in Table 2. SDF was composed of mannose, rhamnose, glucuronic acid, galacturonic acid, arabinose, and fucose in a concentration ratio of 3.56:2.03:19.28:18.55:27.59:29.00, respectively; whereas galactose and xylose were not detected. IDF consisted of mannose, rhamnose, glucuronic acid, galacturonic acid, galactose, xylose, arabinose, and fucose in a ratio of 1.54:1.83:14.01:9.86:29.26:9.40:24.33:9.77, respectively. Arabinose was abundant in monosaccharides in both SDF and IDF, indicating that arabinose is conducive to hemicellulose (28).

**Table 2:** Monosaccharide composition of SDF and IDF.

**%**	**SDF**	**IDF**
Mannose	3.56	1.54
Rhamnose	2.03	1.83
Glucuronic acid	19.28	14.01
Galacturonic acid	18.55	9.86
Galactose	ND	29.26
Xylose	ND	9.40
Arabinose	27.59	24.33
Fucose	29.00	9.77

Previous research has demonstrated that an arabinose- and rhamnose-containing polysaccharides may lower blood glucose levels in diabetic mice, and that arabinose supplementation can lessen glucose peaks and postpone insulin peaks (16). However, in IDF, xylose was the most prevalent monosaccharide, suggesting that there may be hemicelluloses with a xylan-type structure, similar to previous work (18). Therefore, the bergamot DF component was also supported by the abundant positive nutritional effects on glucose regulation in the human body.

### *In vitro* analysis of WHC, OHC, and SC

#### WHC

**Figure 3:**
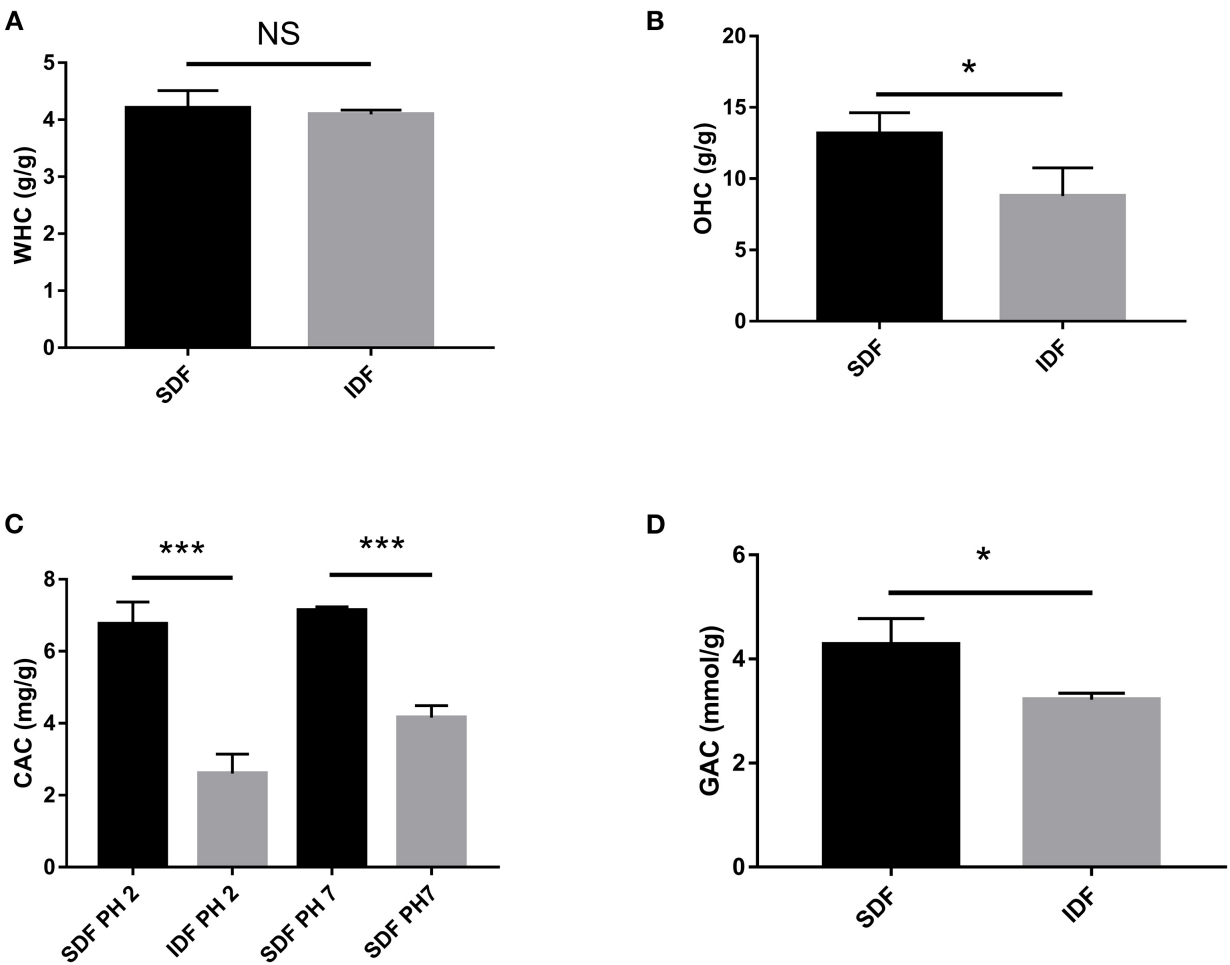
*In vitro* effect of the WHC **(A)**, OHC **(B)**, CAC **(C)**, and GAC **(D)** of SDF and IDF. Each value represents the mean ± SD. The results were analyzed with the two-tailed Student's *t-*test; *p < 0.05, ***p < 0.001, NS: non-significant.

Due of its uneven, loose, and porous surface, DF has a high WHC (29). After absorbing water, DF also has a lubricating effect that can stimulate intestinal peristalsis and motility by fermenting to create short chain fatty acids produced from the gut microbiota that prevent diabetes (29). Figure 3A shows that the WHC of SDF and IDF was 4.21 ± 0.30 g/g and 4.09 ± 0.08 g/g, respectively. Similarly, DF from pomelo peel, with a honeycomb-like structure, has a WHC (23). The WHC of SDF was better than that in IDF group. This phenomenon may contribute to SDF having a porous structure to capture water, which could imply that SDF holds water *in vitro* and could be utilized by the gut microbiota in the intestinal micro-environment to prevent diabetes.

#### OHC

The average charge density, hydrophobicity, physical properties, and preparation procedures all influence the OHC of DF (29). The OHC of SDF was higher than the IDF group (*p* < 0.05, Figure 3B). It was shown that the OHC of carrot DF was greatly enhanced due to the enzymatic hydrolysis exposing its cellular structure (30). Therefore, enzymatic treatment of bergamot DF could enhance its OHC. Moreover, the OHC of DF has important effects for food applications and maintaining human health by adsorbing fats from food and the digestive tract to prevent the development of hyperlipidemia (23). Therefore, SDF could potentially mitigate obesity.

#### CAC

The CAC of DF is classified as either physical or chemical adsorption. Physical adsorption is primarily affected by DF particle size, porosity, and temperature, whereas chemical adsorption is influenced by DF charge and hydrophobic groups (29). The CAC was evaluated at two different pH levels, 2.0 and 7.0, to model cholesterol adsorption in the gastrointestinal system and small intestine environments, respectively (31). Figure 3C shows the different adsorption capacities of SDF and IDF for cholesterol at pH 2.0 and 7.0. At the same pH level, the adsorption capacity of SDF (6.76 ± 0.61 and 7.14 ± 0.10 mg/g) to cholesterol was significantly higher than that of IDF (2.60 ± 0.54 and 4.16 ± 0.33 mg/g). SEM analysis showed that the SDF had a honeycomb-like reticular structure. The surface and structural characteristics of SDF enable it to bind cholesterol molecules through adsorbing and chemical chelation at the physical level, which is consistent with previous work (32). Therefore, SDF and IDF could balance cholesterol levels, and SDF showed a better CAC.

#### GAC

The physiological properties of DF include GAC, reflecting the effect of DF on blood glucose control (33). As shown in Figure 3D, SDF showed a stronger glucose adsorption capacity than IDF (*p* < 0.05). Because glucose may attach to the DF network structure and limit interaction with the human digestive tract to produce a physical barrier and delay glucose diffusion, the GAC of DF is connected to its physical features (29). This supports our result that the SDF microstructure is reticular and absorbs more glucose. The results predicted that SDF had a positive effect in preventing postprandial hyperglycemia.

### Effects of DF on BW and blood glucose levels

**Figure 4:**
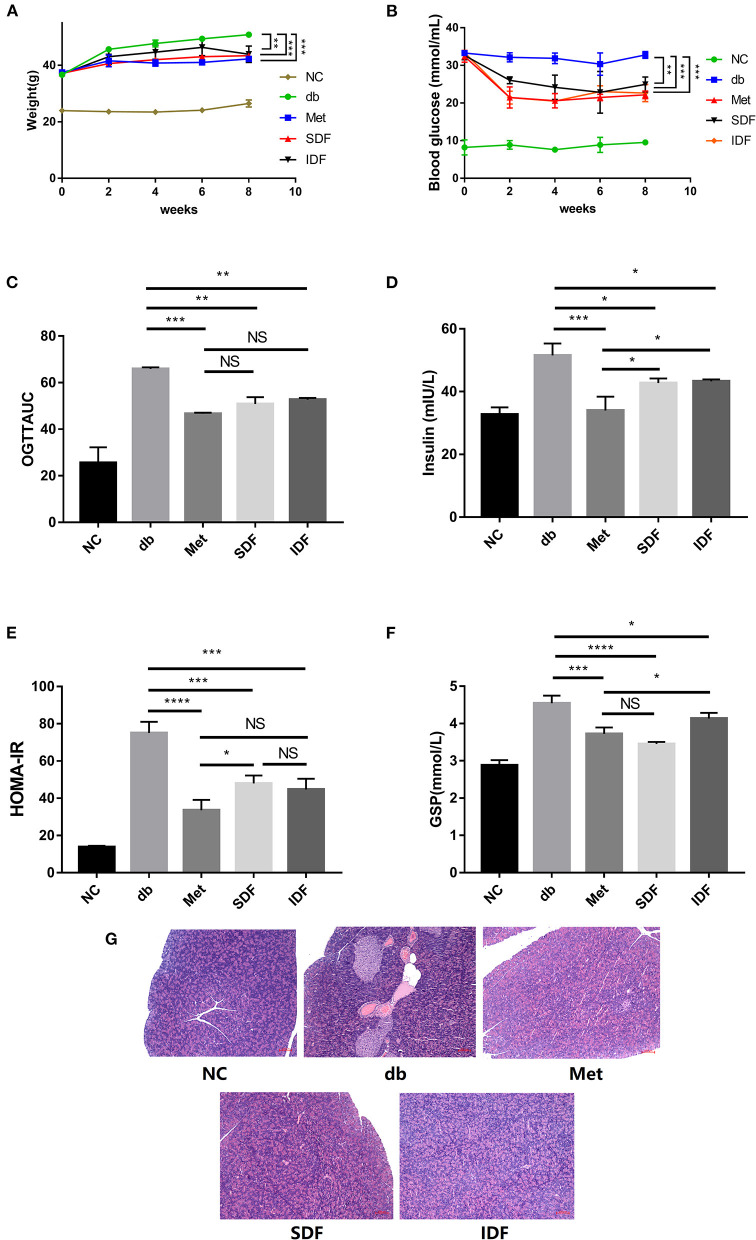
DF relieves glucose intolerance in db/db mice. The **(A)** BW, **(B)** blood glucose, and **(C)** OGTT results of mice. **(D)** Insulin concentration changes were recorded at the indicated times. **(E)** The HOMA-IR scores of the mice were calculated at the end of the study. **(F)** The level of GSP was determined. **(G)** Microscopic images of pancreas sections. Pancreas sections were stained by H&E. Bar = 100 μm. Each value represents the mean ± SD. The results were analyzed via the one-way ANOVA with a Tukey *post-hoc* test; *p < 0.05, **p < 0.01, ***p < 0.001, ****p < 0.0001, NS, non-significant.

Because db/db diabetic mice are functionally defective in the long-form leptin receptor, they display obesity, spontaneous persistent hyperglycemia, and hyperlipidemia (34). The BW of mice was measured every two weeks. The BW of db/db mice rose more dramatically than that of the other groups. However, the BW gain of SDF-treated (6.20 ± 0.58 g) and IDF-treated (7.03 ± 2.62 g) mice was less than that of the db group (14.17 ± 0.17 g). Furthermore, there was a significant difference between the SDF (*p* < 0.001) and IDF groups (*p* < 0.01) compared with the db group (Figure 4A). These data indicate that DF can relieve the symptoms of obesity in db/db mice.

For eight weeks, the blood glucose level in db/db mice was high, compared with that in the other treatment groups (Figure 4B), indicating that the db/db mice were hyperglycemic. Meanwhile, following met intervention, blood glucose levels were considerably lower at 8 weeks compared to db/db vehicle mice (*p* < 0.001). Specifically, blood glucose levels in the SDF and IDF groups fell to 24.93 ± 1.99 and 22.63 ± 2.27 mmol/mL, respectively, and were lower than the values in the db group (32.77 ± 0.90 mmol/mL). The db/db mice exhibit severe symptoms of hyperglycemia (34). In this study, SDF and IDF slowed weight accumulation and lowered blood glucose levels in mice. Similar reports with DF from soy showed a reduction in postprandial hyperglycemia (35). Overall, this indicated that dietary interventions with SDF and IDF may positively affect BW and blood glucose levels in db/db diabetic mice, which may prevent hyperglycemia symptoms caused by leptin receptor disruption.

### Glucose tolerance

The oral glucose tolerance test is an effective and easy way to detect impaired glucose metabolism, and the findings are more precise than fasting blood glucose values alone and can also be used to evaluate pancreatic β-cell function and glucose metabolism (36).

As shown in Figure 4C, the AUC values of db/db diabetic mice were considerably greater than those of the met group (*p* < 0.001). Conversely, unlike in db/db diabetic mice, the AUC values of SDF and IDF groups from the OGTT in db/db diabetic mice decreased significantly (*p* < 0.01).

Insulin resistance is a defining feature of T2DM. The homeostatic index of insulin resistance (HOMA-IR) and insulin levels are shown in Figures 4D,E. Insulin levels were considerably lower after SDF and IDF treatment compared to the db group (*p* < 0.05). In the SDF group, the insulin level was 43.29 ± 0.56 mIU/L, lower than that of the IDF group (44.37 ± 1.91 mIU/L); however, the difference was not significant (Figure 4D).

The increases in HOMA-IR in each group exhibited a similar tendency to the changes in insulin levels. The metformin group showed a significant decrease in insulin level and HOMA-IR (*p* < 0.0001) values, as opposed to the db group, demonstrating that metformin has a positive impact in decreasing insulin resistance. In contrast, HOMA-IR in the SDF and IDF groups was significantly decreased compared to that in the db group (*p* < 0.001), with no significant difference (Figure 4E).

One of glucoregulatory mechanisms of DF has been shown to increase insulin sensitivity in a variety of cells. Soluble fiber slows postprandial glucose absorption by prolonging digestion time, resulting in balanced glucose and insulin levels (9). These results revealed that the uptake of SDF and IDF can enhance the glycemic regulation ability of db/db diabetic mice and enhance insulin sensitivity in target organs to regulate glucose homeostasis.

### Effects of DF on GSP levels

The GSP value is a diabetes indicator that shows the level of blood glucose control (16). The db/db group had significantly greater GSP content than the metformin group. Compared with the GSP levels in the db/db group, those in the SDF and IDF groups decreased. No significant differences were observed between the metformin and SDF group. In addition, no significant differences were observed between the SDF and IDF groups (Figure 4F). These results indicate that intervention with SDF and IDF can reduce the level of GSP in diabetic mice.

### Pathology analysis of pancreas

Insulin resistance is a pathophysiological state in which cells or tissues are resistant to insulin activity and is the primary pathogenic cause leading to T2DM (21). Additionally, T2DM in db/db mice is characterized by insulin resistance, followed by pancreatic β-cell loss (37). The pancreatic structure of db/db mice was disrupted, according to histological analysis of degenerative alterations in the pancreas, and the islet cells had an irregular shape, blurred contour, uneven distribution, and decreased amount.

After treatment with metformin, SDF, and IDF, islet necrosis and pancreatic β-cell injury were significantly reversed. Furthermore, the islet tissue structure was largely intact, and the islet cells were clearer, with uniform distribution and increased numbers (Figure 4G). These results indicated that SDF and IDF could ameliorate damage to pancreatic tissue and restore the pancreatic islet cells in diabetic db/db mice.

### Effect of DF on blood lipid reduction and liver protection in hyperglycemic mice

**Figure 5:**
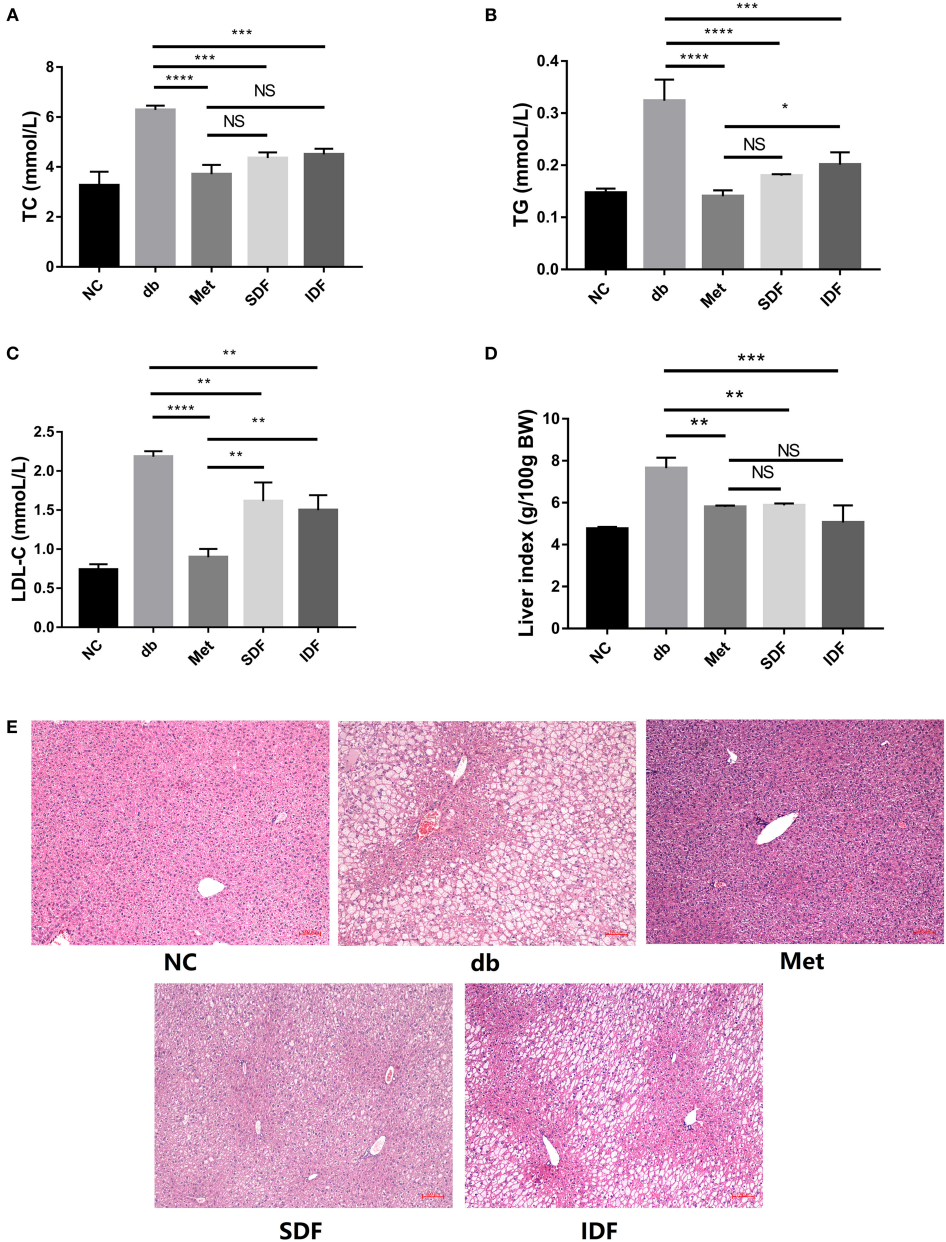
DF improved glycolipid metabolism in db/db mice. Serum levels of TC **(A)**, TG **(B)**, and LDL-C **(C)** were analyzed by commercial kits at the end of the study. Liver index of the db/db mice **(D)**. Microscopic images of liver sections **(E)**. Liver sections were stained by H&E. Bar = 100 μm. Each value represents the mean ± SD. The results were analyzed using one-way ANOVA and a Tukey *post-hoc* test; *p < 0.05, **p < 0.01, ***p < 0.001, ****p < 0.0001, NS: non-significant.

To study the hypolipidemic impact of SDF and IDF on diabetic db/db mice, blood levels of TC, TG, and LDL-C were measured. The TC levels in the db group were significantly greater than in the metformin group (*p* < 0.0001). Furthermore, compared to levels in the db group, TC was dramatically lowered (*p* < 0.001) in mice subjected to SDF and IDF treatment (Figure 5A). When compared to diabetic db/db mice, SDF and IDF dramatically lowered serum TG levels (Figure 5B). The metformin and SDF groups observed no significant differences in TG levels.

The LDL-C level was significantly higher in the db group than in the metformin group (*p* < 0.0001). LDL-C levels significantly decreased in mice treated with SDF (*p* < 0.01) and IDF compared to those in the db group (*p* < 0.01) (Figure 5C). These findings revealed that SDF and IDF had the effect of glycolipid metabolism mitigation.

The body obtains metabolic energy by producing glucose via hepatic glycogen disintegration and converting free fatty acids generated by adipose tissue deterioration (16). When the rate of sugar consumption is slowed, fatty acids become the primary energy source, reducing intracellular glucose utilization and exacerbating diabetic symptoms (16). When the pace of sugar consumption slows, fatty acids become the predominant source of energy, lowering intracellular glucose utilization and worsening diabetes symptoms (16). Glucose and lipid metabolism are linked in a variety of ways, with dyslipidemia in diabetes patients being the most prominent clinical manifestation (21). In our work, blood lipid markers such as TC, TG, and LDL-C were dramatically lowered in diabetic db/db mice following SDF and IDF therapy (Figures 5A–C), indicating that SDF and IDF could regulate the blood lipid contents by mitigating the levels of TC, TG, and LDL-C.

However, the liver index of mice in db/db group was significantly higher (*p* < 0.01) than that of the metformin group (Figure 5D), whereas the liver index of diabetic db/db mice was significantly reduced after SDF (*p* < 0.01) and IDF (*p* < 0.001) intervention. Excessive blood glucose may be the cause of fat accumulation in the liver and may be linked to the formation of dyslipidemia (20). Furthermore, hepatic lipid accumulation is highly associated with the severity of insulin resistance (21). As shown in Figure 5E, histological analysis revealed that the liver structure was not manifested in neatly arranged hepatic cords, and prominent cell vacuolization was evident in db/db mice. However, compared to hepatocytes in the db group, the hepatocytes in the metformin, SDF, and IDF groups had a prominent structure, uniform size, and orderly arrangement, and the degree of vacuolization was mild and relieved; in particular, the hepatocytes reverted to their normal state. Histological liver tests indicated that pathological alterations in diabetic db/db mice included evident cell vacuolization and substantial lipid droplet accumulation; however, SDF and IDF therapy significantly reduced these symptoms. As a result, the downregulation of dyslipidemia in liver tissues by SDF and IDF may be partially responsible for the improvement in hepatic insulin resistance in diabetic db/db mice.

## Conclusions

Diabetes and its complications are becoming increasingly common worldwide, seriously influencing the lives of human beings. It is a prevalent chronic metabolic condition defined by dysglycemia and dyslipidemia, both of which inevitably result in health issues such as eye, kidney, heart, and hepatic dysfunction. This means that maintaining blood glucose and lipid levels is vital for diabetes treatment and prevention.

In this study, the SDF and IDF were extracted from bergamot. Their chemical composition, structural characterization, physicochemical properties, and their effects on blood glucose control and lipid levels in db/db mice were investigated. The DF network structures are completely different. SDF is loose and has honeycomb-like holes and cracks, while IDF is flaky and schistose with a smooth surface and dense texture. In terms of monosaccharide composition, arabinose was abundant in monosaccharides in both SDF and IDF. SDF and IDF from bergamot exhibited different structures, which varied in their WHC, OHC, CAC, and GAC *in vitro*. Notably, these capabilities of SDF were comparatively more effective owing to its loose and polar structure to bind with oil, cholesterol, and glucose. SDF and IDF mitigate the symptoms of hyperglycemia and protect pancreatic tissue. Specifically, SDF can enhance the glycemic regulation ability of db/db diabetic mice and may enhance insulin sensitivity in the target organs to regulate glucose homeostasis. *In vivo* experiments also indicated that SDF and IDF effectively controlled blood lipid levels and protected liver tissue in db/db diabetic mice. Also, this study discovered that SDF can more effectively inhibit cholesterol micelle production *in vitro* and control blood lipid levels *in vivo* by lowering serum TC, TG, and LDL-C levels, which is important for cardiovascular health and body shape, particularly when consuming fried or fatty meals.

Although dysfunction of db/db diabetes mice is complicated in its physiology, our future work will explore its pathway and elucidate the regulatory mechanism of bergamot SDF and IDF. Overall, the outcomes of this study show that bergamot SDF and IDF might be used to create and test multivalent functional foods that could supplement existing dietary interventions.

## Data availability statement

The original contributions presented in the study are included in the article/supplementary material, further inquiries can be directed to the corresponding author.

## Ethics statement

The animal study was reviewed and approved by Hangzhou Laboratory Animal Operation Center of Yanxuan Biotechnology Company. Written informed consent was obtained from the owners for the participation of their animals in this study.

## Author contributions

JLi and HL: conceptualization and methodology. JLi: data curation and writing—original draft preparation. GL, JLa, and RZ: visualization and investigation. GX: supervision. CL: software and validation. QW and JLi: writing—reviewing and editing. All authors contributed to the article and approved the submitted version.

## Funding

This work was supported by Research and Application on Germplasm Innovation and New Variety Cultivation Technology of Subtropical Fruits with Characteristics in Western Guangdong and Key areas of Guangdong Province Research and Development Plan project in 2021.

## Conflict of interest

The authors declare that the research was conducted in the absence of any commercial or financial relationships that could be construed as a potential conflict of interest.

## Publisher's note

All claims expressed in this article are solely those of the authors and do not necessarily represent those of their affiliated organizations, or those of the publisher, the editors and the reviewers. Any product that may be evaluated in this article, or claim that may be made by its manufacturer, is not guaranteed or endorsed by the publisher.

## Abbreviations

DF: Dietary fiber
SDF: Soluble dietary fiber
IDF: Insoluble dietary fiber
T2DM: Type 2 diabetes mellitus
FT-IR: Fourier-transform infrared spectroscopy (FT-IR)
PMP: 1-phenyl-3-methyl-5-pyrazolone
WHC: Water-holding capacity
OHC: Oil-holding capacity
CAC: Cholesterol adsorption capacity
GAC: Glucose adsorption capacity
BW: Body weight
FBG: Fasting blood glucose
GSP: Glycated serum protein
HOMA-IR: Homeostatic index of insulin resistance
OGTT: Oral glucose tolerance test
TC, Total cholesterol, TG: Triglycerides
LDL-C: Low-density lipoprotein cholesterol
SD, Standard deviation, Met: Metformin.
